# Detection of maxillary sinus fungal ball via 3-D CNN-based artificial intelligence: Fully automated system and clinical validation

**DOI:** 10.1371/journal.pone.0263125

**Published:** 2022-02-25

**Authors:** Kyung-Su Kim, Byung Kil Kim, Myung Jin Chung, Hyun Bin Cho, Beak Hwan Cho, Yong Gi Jung

**Affiliations:** 1 Medical AI Research Center, Samsung Medical Center, Seoul, Republic of Korea; 2 Department of Otorhinolaryngology-Head and Neck Surgery, Samsung Medical Center, Sungkyunkwan University School of Medicine, Seoul, Republic of Korea; 3 Department of Radiology, Samsung Medical Center, Sungkyunkwan University School of Medicine, Seoul, Republic of Korea; 4 Department of Medical Device Management and Research, SAIHST, Samsung Medical Center, Sungkyunkwan University School of Medicine, Seoul, Republic of Korea; University Tunku Abdul Rahman, MALAYSIA

## Abstract

**Background:**

This study aims to develop artificial intelligence (AI) system to automatically classify patients with maxillary sinus fungal ball (MFB), chronic rhinosinusitis (CRS), and healthy controls (HCs).

**Methods:**

We collected 512 coronal image sets from ostiomeatal unit computed tomography (OMU CT) performed on subjects who visited a single tertiary hospital. These data included 254 MFB, 128 CRS, and 130 HC subjects and were used for training the proposed AI system. The AI system takes these 1024 sets of half CT images as input and classifies these as MFB, CRS, or HC. To optimize the classification performance, we adopted a 3-D convolutional neural network of ResNet 18. We also collected 64 coronal OMU CT image sets for external validation, including 26 MFB, 18 CRS, and 20 HCs from subjects from another referral hospital. Finally, the performance of the developed AI system was compared with that of the otolaryngology resident physicians.

**Results:**

Classification performance was evaluated using internal 5-fold cross-validation (818 training and 206 internal validation data) and external validation (128 data). The area under the receiver operating characteristic over the internal 5-fold cross-validation and the external validation was 0.96 ±0.006 and 0.97 ±0.006, respectively. The accuracy of the internal 5-fold cross-validation and the external validation was 87.5 ±2.3% and 88.4 ±3.1%, respectively. As a result of performing a classification test on external validation data from six otolaryngology resident physicians, the accuracy was obtained as 84.6 ±11.3%.

**Conclusions:**

This AI system is the first study to classify MFB, CRS, and HC using deep neural networks to the best of our knowledge. The proposed system is fully automatic but performs similarly to or better than otolaryngology resident physicians. Therefore, we believe that in regions where otolaryngology specialists are scarce, the proposed AI will perform sufficiently effective diagnosis on behalf of doctors.

## 1 Introduction

Maxillary sinus fungal ball (MFB) is a common cause of unilateral chronic maxillary sinusitis and is a condition that requires surgical treatment because it causes severe mucosal inflammation and does not respond to medical treatment. The fungal ball on computed tomography (CT) shows characteristic features such as intralesional calcification, the spiculated surface of soft tissue density, complete opacification of the sinus cavity, and bony wall thickening involving the sinus [1, 2]. A well-trained otolaryngologist or radiologist can distinguish between chronic maxillary sinusitis without a fungal ball, which may respond to medical treatment, and MFB requiring surgery by understanding the radiological characteristics of MFB.

Recently, CT scans, including cone-beam CT, have become popular and are frequently used in primary care clinics [1]. However, a primary care provider who is not familiar with sinus imaging may miss the diagnosis of a fungal ball requiring surgery, and they might try medical treatment for MFB. Therefore, there is a need to provide primary care doctors with a tool that can help them to make a correct diagnosis if they are not experts in this field.

As a solution to these problems, artificial intelligence (AI) and deep learning are increasingly being used in the medical image-based analysis [2]. Among them, convolutional neural networks (CNNs) are a widely used deep learning method tool. Some studies have used CNN techniques for CT-based image analysis in the area of paranasal sinuses and nasal cavities [3–5]. In previous studies, osteomeatal complex inflammation, anterior ethmoidal artery location, and middle turbinate pneumatization were classified by a trained 2-dimensional convolutional neural network (2-D CNN) using a single slice of coronal CT image. However, 2-D CNN essentially has disadvantages in terms of the 3-D context of original images and may not reflect the real condition of the patient. To further increase the accuracy of this technology and its clinical application, it would be better to use a 3-dimensional CNN (3-D CNN) that has employed the entire section of the CT images. As Huang *et al*. reported, 3-D CNNs were more sensitive than 2-D CNNs for analyzing and detecting lung nodules from a stereoscopic perspective [6].

Herein, we propose an AI system that uses 3-D CNN techniques to automatically classify MFB, non-fungal maxillary sinusitis (i.e., chronic rhinosinusitis, CRS), and normal maxillary sinus (i.e., healthy control, HC). We will also evaluate the accuracy of the trained 3-D CNN model by internal and external validation and compare its performance with that of otolaryngology resident physicians.

## 2 Methods

In this section, we introduce the proposed AI technology (Section 2.3). Then, the data collection and labeling process (Section 2.1) and novel data preprocessing (Section 2.2) are shown.

### 2.1 Data acquisition and annotation

We collected internal and external datasets from Samsung Medical Center, Gangnam-Gu, Seoul, Korea, and Samsung Changwon Hospital, MasanHoiwon-gu, Changwon-si, Gyeongsangnam-do, Korea, respectively, after approval by the Institutional Review Board of Samsung Seoul Hospital (SMC 2020-07-173). In other words, the internal and external datasets were collected from different hospitals, and they consisted of different patients. The internal dataset (n = 512) was captured from 425 General Electric, 37 Toshiba, 31 Siemens, and 19 Philips CT scanners. The external dataset (n = 64) was obtained from 54 Siemens, 11 Philips, and 1 Toshiba scanners. Each scanner has the same imaging parameters for 120 kVP and 2mm of slice thickness in the coronal plane. All Digital Imaging and Communications in Medicine (DICOM) data of CT scans were preprocessed in a homogenous course to minimize differences between scanners. Hounsfield Unit (HU) was used to measure the radiodensity of CT scans, and finally, it was converted into voxel values for training and validation. For image contrast enhancement, we also restricted the upper and lower gray levels of DICOM data by setting the window level and window width to 0 and 2000, respectively. We used these values as recommended to optimize the observation of fungal balls [7]. After passing the filter, all images were rescaled with a consistent image size of 512 × 512 and were normalized for each image to range from 0 to 255.

**Fig 1:**
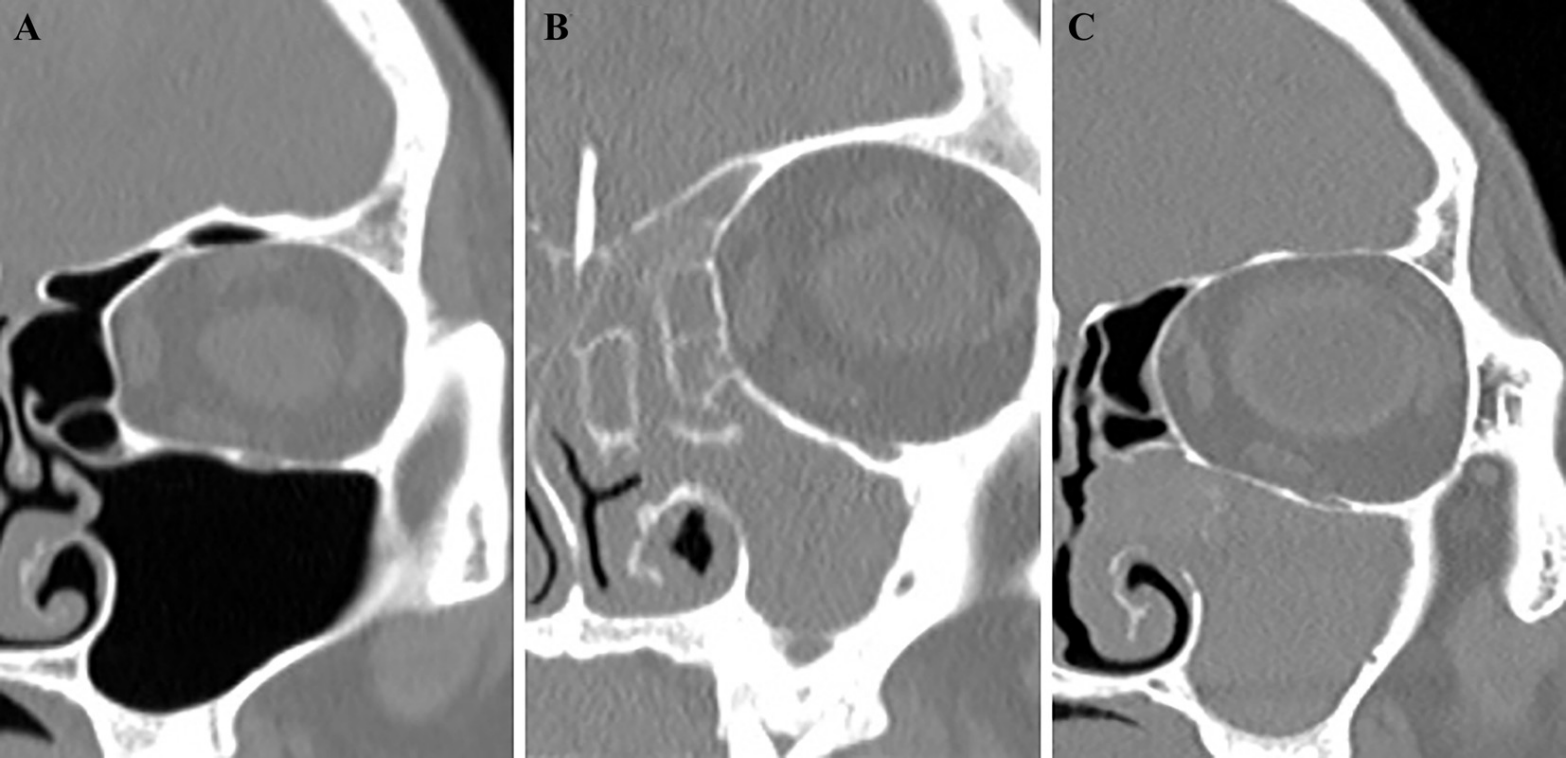
Coronal computed tomography images of representative cases with (A) healthy control, (B) chronic rhinosinusitis, and (C) maxillary sinus fungal ball on half coronal CT slices.

The internal dataset included 512 3-D stacks of ostiomeatal unit computer tomography (OMU CT) images of the coronally observed entire head. The manifestation of a target disease in a single 3-D stack consisted of 254 patients with MFB, 128 patients with CRS, and 130 HCs. Similarly, we collected an external dataset from 26 patients with MFB, 18 patients with CRS, and 20 HCs. Fig 1 shows an example of each of MFB, CRS, and HC.

All MFBs included in this study were patients with pathologically proven fungal material through previous endoscopic sinus surgery. In this process of annotating the other two classes (i.e., CRS and HC) for each 3-D hemi-stack for the left or right side of OMU CT slices (i.e., annotating one of the three classes according to whether the corresponding symptom presented at each of both sides of the maxillary sinus), five experts, four otolaryngologists with less than ten years of experience, and one otolaryngologist with more than ten years of experience were individually asked to label each class on both sides of the maxillary sinus. Afterward, they discussed their evaluations to arrive at one common opinion per stack, supported by three or more experts.

### 2.2 Data preprocessing through division

For both datasets, it is helpful to note that most of the patients with MFB had the disease on only one side of their left and right maxillary sinuses. Therefore, if one side of the maxillary sinus has a disease (i.e., MFB or CRS) but the other side does not have it, this 3-D hemi-stack without the disease can be considered HC.

Owing to this feature, we cut each original 3-D stack of the head CT set in half and generated two 3-D hemi-stacks for the right and left head voxels. Each 3-D hemi-stack was created with a single (i.e., left or right) maxillary sinus located in the center of this 3-D image, whereas the original 3-D stack before the division showed both maxillary sinuses. Accordingly, as the central focus of the hemi-stack was on the entire interior of the maxillary sinus, through the division, we were able to diagnose the presence or absence of diseases in each maxillary sinus by letting AI scan the entire interior of the maxillary sinus at each 3-D hemi-stack to classify MFB, CRS, and HC.

Through this division, the number of 3-D stacks for MFB, CRS, and HC subjects in the internal dataset increased from 254, 128, and 130 to 266, 256, and 502, respectively. Using the same method, the number of 3-D stacks for MFB, CRS, and HC subjects in the external dataset increased from 26, 18, and 20 to 27, 32, and 69, respectively. In other words, the number of total images for training the AI-based system increased from 512 to 1024; in particular, the number of HCs increased by approximately four times from 130 to 502. This increase in HCs provided additional AI information for the healthy control group, allowing the AI to differentiate between abnormal and normal subjects more effectively. Tables 1 and 2 summarize the number of internal and external data sets of whole and half 3-D stacks, respectively.

**Table 1:** The number of 3-D full-stacks (patients) of OMU CT images for each class in the internal and external dataset (total n = 512 and 64 respectively).

Subjects	Internal dataset, *n* (%)	External dataset, *n* (%)
MFB	254 (49.6)	26 (40.6)
CRS	128 (25.0)	18 (28.1)
HC	130 (25.4)	20 (31.3)

**Table 2:** The number of 3-D hemi-stacks of OMU CT images for each class in the internal and external dataset (total n = 1024 and 128 respectively).

Subjects	Internal dataset, *n* (%)	External dataset, *n* (%)
MFB	266 (26.0)	27 (21.1)
CRS	256 (25.0)	32 (25.0)
HC	502 (49.0)	69 (53.9)

### 2.3 Overview of the proposed algorithm

**Fig 2:**
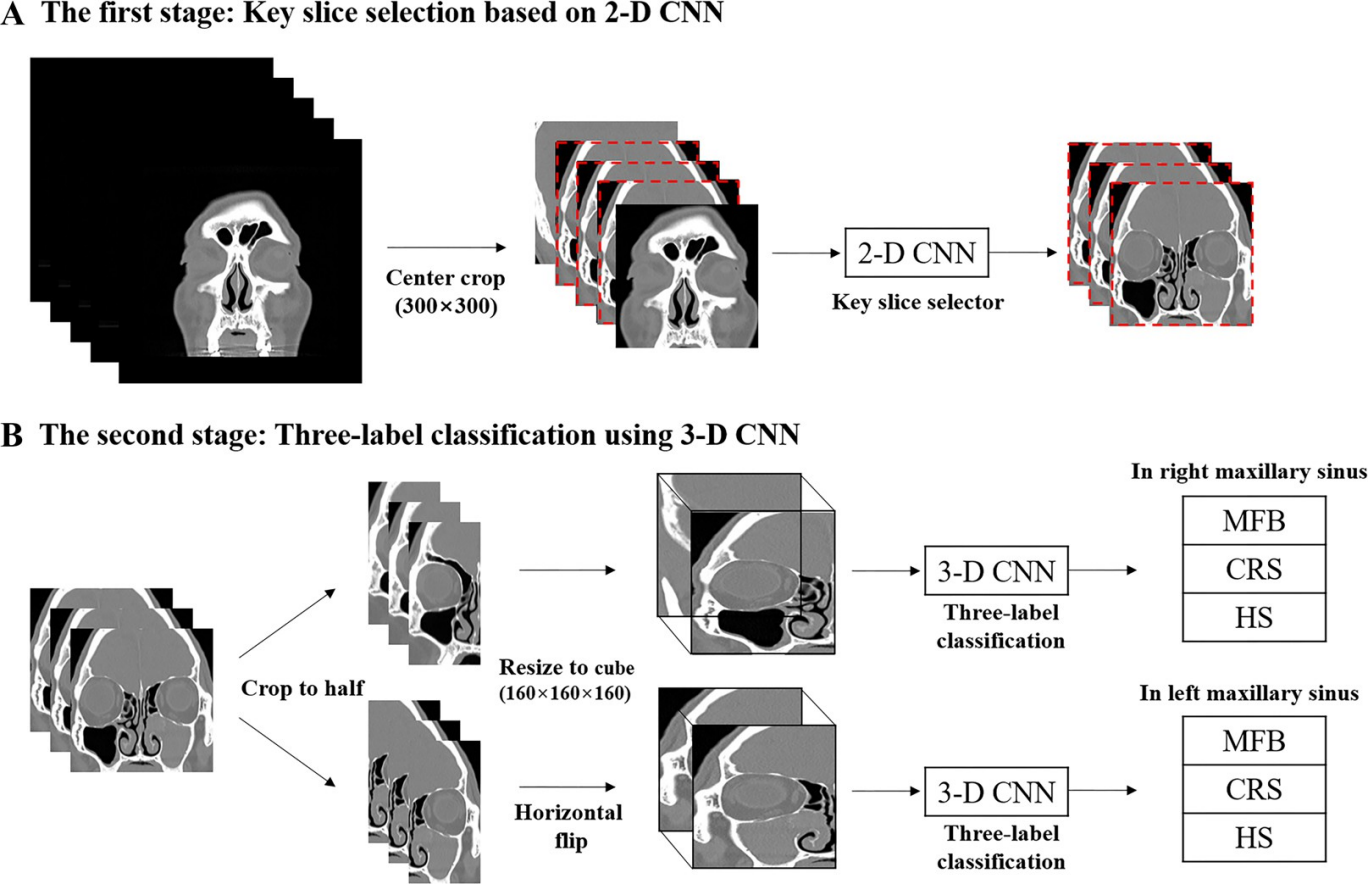
Overview of the proposed network algorithm. In the first stage (A), key slices were automatically extracted from the entire section of the coronal CT image using the 2D-CNN technique. In the second stage (B), disease classification was performed through the 3D-CNN by taking a 3D stack composed of only key CT slices as input. MFB; maxillary sinus fungal ball, CRS; chronic rhinosinusitis, HC; healthy control, CNN; convolutional neural network.

**Fig 3:**
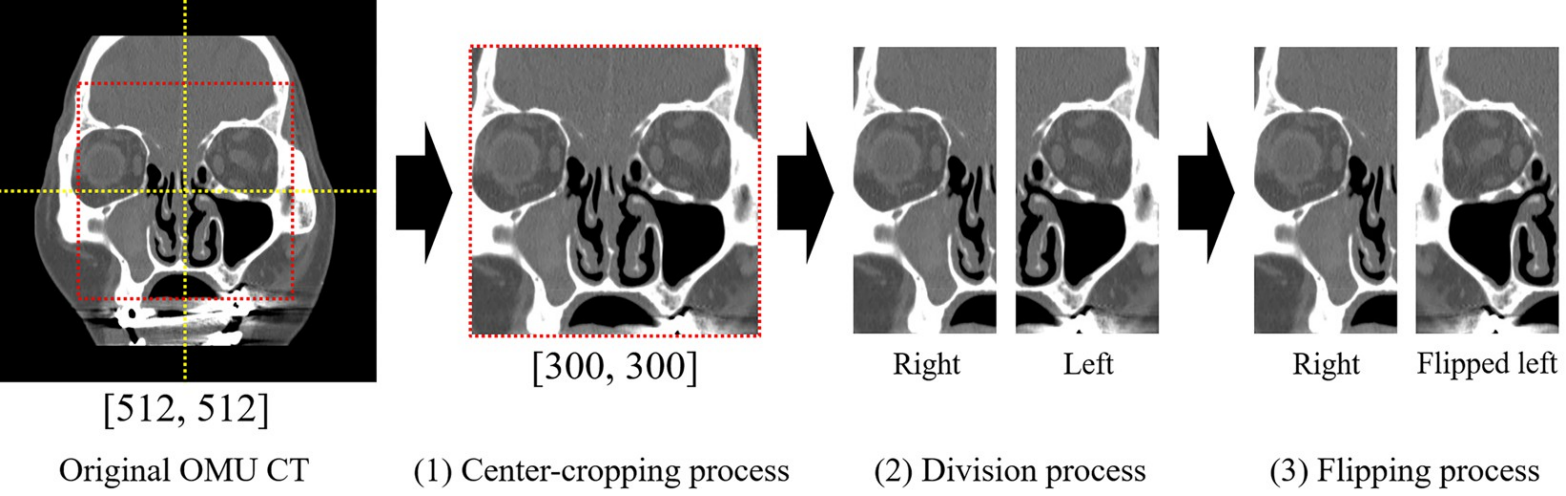
Illustration of cropping, division, and flipping processes.

In this section, we describe the proposed AI-based classifier, whose overall architecture is illustrated in Fig 2. The proposed algorithm consists of two steps with a fully automated process using the entire stack of OMU CT slices; the first step is for a 2-D CNN to select a subset of coronal slices, including the maxillary sinus from the whole OMU CT stack. We performed this process to improve the classification performance by focusing only on the maxillary sinus part of the entire CT stack with limited training data. This step is shown in Fig 2A. We selected approximately 20 coronal slices with the maxillary sinus from various numbers of full-stack slices (n = 40–100). In the second step, as shown in Fig 2B, we used a 3-D CNN to classify the MFB, CRS, and HC subjects at each of the left and right maxillary sinuses. Given the coronal sub-slices selected by the first step, we split into left and right hemi-slices as described in Section 2.2, horizontally flipped the left-sided hemi-slices to the right-sided ones, and took this right-sided 3-D stack of hemi-slices as an input of the 3-D CNN. We illustrated these processes in Fig 3. These splitting and flipping processes doubled the amount of training dataset, thereby improving the classification performance of AI. We used a high-performance system, including an Intel Core i7-7700 CPU and NVIDIA GeForce GTX 1080 Ti GPU, to train and test our two-stage algorithm. All image processing and deep learning were performed using Pytorch 1.6 with Python 3.6.

#### 2.3.1 The first-stage sub-algorithm: Key-slice selector based on neural network

Because MFB and CRS exist in the maxillary sinus if only the coronal slices of the 3-D voxel for the entire head can be automatically and successfully selected, the ability to detect abnormalities can be improved. In this sense, as the first step of the proposed AI framework, we used a 2-D CNN and made it fully automatically extract sub-slices belonging to the maxillary sinus region among a 3-D stack of OMU CT whole slices.

As a key-slice selector, this CNN model scanned each coronal slice and provided a value of 1 if the maxillary sinus was visible on the slice and 0 otherwise. To train the network to perform this binary classification task, the five otolaryngologists mentioned previously had annotated whether the maxillary sinus was visible in each coronal slice of each patient’s head 3-D stack among the internal dataset of CRS and HC (i.e., marking 1 or 0 at each coronal hemi-slice). This annotated training dataset consisted of 258 (i.e., 128 for CRS and 130 for HC) 3-D stacks of whole slices, where each slice was annotated by 1 or 0 according to the presence or absence of maxillary sinus, respectively, and was used to train the 2-D CNN network to predict the annotation value (i.e., 1 or 0) and discriminate the slice with or without the presence of maxillary sinus.

We utilized EfficientNet as the backbone of our 2-D CNN [8]. To prevent issues of over-fitting and vanishing gradient, we selected the smallest model (i.e., EfficientNet-b0) among various EfficientNet models (i.e., from EfficientNet-b0 to -b7) [9]. We applied center cropping to 300 × 300 at all slices to eliminate unnecessary space. In addition, as the original CT slice has 1-channel, we duplicated it to create 3-channel images for the network input and applied the model’s weight pre-trained by ImageNet to our model’s initial weight.

We trained the neural network for 30 epochs with a mini-batch size of 256. An Adam optimizer with a cosine annealing scheduler from an initial learning rate of 10^−5^ was used (Ref Adam, Ref cosine). Binary cross-entropy was used as the loss function in training. We computed the probability using sigmoid activation at the outputs of the 2-D CNN, where the binary classification criteria for key-slice estimation were set to 0.5.

#### 2.3.2 The second-stage sub-algorithm: Classification using 3-D CNN

Unlike 2D CNN, the 3D CNN additionally uses information on adjacent associations between slices to effectively detect subtle differences in disease expression phenomena caused by adjacent changes between coronal slices. Based on this fact, we used a 3-D CNN to classify the three cases, including MFS, CRS, and HC subjects. The proposed 3-D CNN takes a right-sided (flipped as left-sided) or left-sided 3-D stack of hemi-slices, each of which was extracted from the key-slice selector of 2-D CNN, as input and provides a predicted value i∈{1, 2, 3} of what the actual class is among MFS, CRS, and HC as one of the values of 1, 2, and 3, respectively.

Each 2-D slice was split into both right and left hemi-slices. The left-sided hemi-slices were then horizontally flipped to the right-sided slices. Finally, we converted the 2-D hemi-slices into 3-D data using area interpolation. The interpolation was performed using the MONAI package (https://monai.io). The height, weight, and thickness of the 3-D data were 160, 160, and 160, respectively, as cubic-like structures. This data size showed the best performance in our study, and cubic data performed well in the 3-D CNN. In addition, we applied various augmentation techniques, such as random shift (±5 pixels) and random scale transformation (±10%) at all axes to overcome the limitation of a small dataset.

As the base model for the 3-D CNN, we adopted ResNet18 [10], in which the initial parameter was pre-trained using the Kinetics dataset [11]. We trained the 3-D CNN for 50 epochs with a mini-batch size of 16. We used the Adam optimizer with a cosine annealing scheduler and a warm-up scheduler. The initial learning rate of the schedulers was 10^−5^. We employed cross-entropy as a loss function for training the 3-D CNN.

### 2.4 Evaluation metrics for classification performance

Classification performance was evaluated according to the following five statistical analyses: accuracy, the area under curve (AUC) for receiver operator characteristics (ROC), sensitivity, precision, and F1 score.

The accuracy is denoted by the percentage of the total number of test samples that CNN actually identifies with the true labels, and the precision and sensitivity are the class-wise averages of the proportions that were detected correctly among all samples detected by the target class and all samples of the target class, respectively. The F1 score is denoted as the harmonic mean of precision and sensitivity. As our task was a multi-label (i.e., three-label) classification, we expressed the MFB, CRS, and HC cases as 1, 2, and 3 and calculated three groups of true positive (TP), false positive (FP), and false-negative (FN) by selecting a target label i∈{1, 2, 3} as positive and the other labels excluding the label as negative.

$$Accuracy=\frac{\sum_{i=1}^{C}T_{i}}{D_{test}}$$

$$Precision=\frac{1}{C}*\sum_{i=1}^{C}{Precision}_{i}=\frac{1}{C}*\sum_{i=1}^{C}\frac{{TP}_{i}}{{TP}_{i}+{FP}_{i}}$$

$$Sensitivity=\frac{1}{C}*\sum_{i=1}^{C}{Sensitivity}_{i}=\frac{1}{C}*\sum_{i=1}^{C}\frac{{TP}_{i}}{{TP}_{i}+{FN}_{i}}$$

$$F\_measure=\frac{1}{C}*\sum_{i=1}^{C}{F\_measure}_{i}=\frac{2}{C}*\sum_{i=1}^{C}\frac{{Precision}_{i}*{Sensitivity}_{i}}{{Precision}_{i}+{Sensitivity}_{i}},$$

where *C* (i.e., 3) is the number of classes, *T_i_* is the number of testing samples with both labels and estimates equal to i∈{1, 2, 3}, *D_test_* is the total number of testing samples, and *TP_i_, FP_i_*, and *FN_i_* denote true positive, false positive, and false negative, respectively, and *Precision_i_, Sensitivity_i_* and *F_measure_i_* denote the precision, sensitivity, and F1 score, respectively, when a label i∈{1, 2, 3} is selected as positive. Then, we calculated the final precision and sensitivity values using their class-wise averages. All statistical analyses were performed using 5-fold cross-validation in the internal dataset. The external dataset was evaluated from five models trained using the internal dataset according to 5-fold cross-validation. We also calculated AUC (ranging from 0 to 1) according to both the micro and macro-average scales (i.e., sample-wise and class-wise averages, respectively).

## 3 Results

In this section, we reported the classification performance of the proposed AI and humans in Sections 3.1 and 3.2 (i.e., internal and external validations, respectively) and supplemented the result in Sections 3.3 and 3.4 (i.e., the effect of slice selector and visualization, respectively).

### 3.1 Cross-validation results on the training set

Before comparing the performance results of AI and humans via external validation, in this section, we first introduced the AI’s performance via internal validation. Then, Table 3 summarizes the AUC and accuracy of AI. As shown in Table 3, the proposed AI algorithm had micro-average and macro-average AUCs of 0.960±0.005 and 0.955 ±0.006 for internal validations, respectively. Accordingly, this observation supports the high diagnostic accuracy and reliability of the proposed AI system in that all fold results show an AUC of 0.95 or more. In addition, as observed with no significant difference between the micro-average AUC and macro-average AUC, our test data were well balanced for each class.

**Table 3:** AUC and accuracy of AI in the internal validation. The average and standard deviation were derived from each of 5-fold cross-validations.

Metrics	Internal validation results
Micro-average AUC of AI	0.960 ±0.005
Macro-average AUC of AI	0.955 ±0.006
Accuracy of AI, %	87.5 ±2.3

As one would naturally expect from these high AUCs of AI, the proposed AI system achieved a high accuracy of approximately 90%. More specifically, in Table 4, the proposed AI system also had a micro-average accuracy of 87.5 ±2.3%.

**Table 4:** Sensitivity, precision, and F1 score of AI in the internal validation. The average and standard deviation in 5-fold cross-validation were denoted.

Dataset	Sensitivity, %			
	Normal	CRS	MFB	Average
Internal	93.0 ±2.3	73.1 ±6.1	91.1 ±3.4	85.7 ±3.9
	Precision, %			
Internal	90.9 ±1.9	80.3 ±5.2	87.8 ±6.6	86.3 ±4.6
	F1 score, %			
Internal	91.9 ±1.5	76.4 ±4.7	89.2 ±3.4	85.8 ±3.2

As shown in Table 4, we also executed the performance evaluation of AI using the sensitivity, precision, and F1 score. By setting each class as positive and the remaining negative, we calculated the sensitivity, precision, and F1 score per class and presented their macro-averages over the classes in the rightmost column. The class-wise averages of these measurements provided more than 85% values, showing similar performance trends to those of the accuracy.

### 3.2 External validation and performance evaluation through comparison with resident physicians

A total of 64 image sets of OMU CT (i.e., 26 MFB, 18 CRS, and 20 HC) were presented to six resident physicians working in the Department of Otolaryngology at Samsung Medical Center, and we asked them to read each image set. This group of majors in otolaryngology was selected from doctors who did not participate in the study or in the study data extraction.

Note that the AI system evaluated in this section is fully automatic. In other words, the proposed AI scheme takes a single OMU 3-D stack without any preprocessing as input, passes it through the aforementioned image preprocessing and the two-stage classification process shown in Fig 2, and executes the three-label classification for each of the left and right maxillary sinuses. We used the five AI models pre-trained using a 5-fold cross-validation internal dataset, unaffected by the external validation dataset. Based on the presence or absence of key-slice detection, the performance comparison, the first stage of our two-stage prediction process, is discussed in Section 3.3.

Before presenting the performance measurements introduced in Section 2.4, we showed a comparison result in the form of a confusion matrix, as all performance measurements were derived from it. We commonly evaluated these results using the external validation data of 64 3-D stacks with OMU CT images. The averages and standard deviations of the proposed AI and human tests were derived from each of the five AI models individually trained by 5-fold cross-validations and each evaluation of the aforementioned six resident physicians, respectively. As shown in Table 5, except for one case (i.e., the case where the actual is CRS and the predicted one is MFB), the proposed AI system predicted better than humans for the other five cases for misclassification. In each of these five error cases, AI had a value of at least 23% less than that of humans. Therefore, we believe that AI has the potential for better or at least comparable performance to that of humans. Furthermore, given that the test subjects were residents who majored in otorhinolaryngology, Table 5 suggests that AI has the potential to make a better diagnosis than non-otolaryngologists.

**Table 5:** Performance comparison between the proposed AI system and resident test. Confusion matrices for external validation data.

Confusion matrix		Predicted			Confusion matrix		Predicted		
		HC	CRS	MFB			HC	CRS	MFB
Actual	HC	64.0 ±2.1	4.0 ±2.1	1.0 ±0.0	Actual	HC	61.1 ±11.4	6.3 ±10.2	1.5 ±1.2
	CRS	0.2 ±0.4	25.2 ±2.4	6.6 ±2.7		CRS	0.7 ±0.5	25.2 ±5.0	6.2 ±5.5
	MFB	1.0 ±0.0	2.0 ±0.7	24.0 ±0.7		MFB	1.3 ±0.5	3.7 ±2.3	22 ±2.3

(a) AI result (Accuracy, 88.4±3.1%).

(b) Resident result (Accuracy, 84.6±11.3%).

Table 6 summarizes the AUCs and accuracies of AI and humans for external validation. As shown in Table 6, the proposed AI algorithm had micro-average and macro-average AUCs of 0.966 ±0.005 and 0.969 ±0.006, respectively. Similarly, with the internal validation result, the proposed AI achieved a high accuracy of approximately 90% and 88.4 ±3.1%. Furthermore, compared to the micro-average accuracy of humans (i.e., 84.6 ±11.3%), as shown in Table 5(B), this result indicates that the proposed AI caused a 2.9% improvement in accuracy and 8% reduction in its standard deviation, demonstrating the usefulness of the proposed AI technology.

**Table 6:** AUC and accuracy of AI and humans in the external validation. The external validation was evaluated from each AI model trained by 5-fold cross-validation or each of six human classification tests, of which average and standard deviation were given.

Metrics	External validation results
Micro-average AUC of AI	0.966 ±0.005
Macro-average AUC of AI	0.969 ±0.006
Accuracy of AI, %	88.4 ±3.1
Accuracy of human residents, %	84.6 ±11.3

To support the rationale for the accuracy improvement, we also present in Table 7 a performance comparison between AI and humans by using the sensitivity, precision, and F1 score. Compared to the class-wise average results of humans, the proposed AI produced a 4.7% (4.7 = 87.6–82.9) improvement in sensitivity, 2.3% (2.3 = 85.2–82.9) improvement in precision, and 3.8% (3.8 = 85.7–81.9) improvement in F1 score. In particular, for the case of sensitivity, AI improved the sensitivity of MFB by 12.6% (12.6 = 89.6–77.0) and the sensitivity of CRS by 5.8% (5.8 = 80.6–74.8) compared to humans. These results help doctors prevent FP diagnosis of the disease (i.e., MFB or CRS).

**Table 7:** Performance comparison between human and AI in the external validation. Sensitivity, precision, and F1 score of each class were given. The average and standard deviation were denoted from each model trained by the 5-fold internal validation.

Subject	Sensitivity, %			
	Normal	CRS	MFB	Average
Human	96.9 ±1.1	74.8 ±18.9	77.0 ±1.3	82.9 ±9.7
AI	92.5 ±1.9	80.6 ±6.0	89.6 ±1.7	87.6 ±3.2
	**Precision, %**			
Human	88.6 ±16.5	78.6 ±15.5	81.5 ±8.4	82.9 ±8.8
AI	98.2 ±0.7	80.9 ±7.1	76.4 ±6.1	85.2 ±4.6
	**F1 score, %**			
Human	91.8 ±10.5	75.8 ±15.8	78.1 ±6.0	81.9 ±10.5
AI	95.4 ±1.7	79.7 ±6.6	82.0 ±3.7	85.7 ±4.0

Comparing Tables 3–6, we observed that the difference in AUC or accuracy between the internal and external validation was less than 2%. Similarly, comparing Tables 4 and 7, we also observed that the average difference of each performance index (i.e., sensitivity, precision, and F1 score) between internal and external validation was less than 2%. These results support the generalizability of the proposed AI system.

Furthermore, to improve the AI performance, we compared the performance of five models trained with 5-fold internal cross-validation as an ensemble model [12]. In other words, we predicted the final label through a majority vote of five label estimates, where they were individually generated from each pre-trained model. In addition, if there were two or more candidate groups, the final label was randomly selected.

Although the ensemble AI model cannot provide the second momentum (i.e., standard deviation) in measuring the accuracy as the five models were integrated into one model, we observed that the ensemble model provided higher accuracy than those individually obtained from each of the five pre-trained AI models. In Table 8, we summarize the performance of AI models with and without considering the ensemble technique in the external validation. These results indicate that the ensemble AI model improved the accuracy by 1.4% and 5.2%, sensitivity by 0.7% and 5.4%, and precision by 2.9% and 5.2% over the individual AI models and the human residents, respectively. Although it cannot be confirmed from our experimental results that the proposed AI model is superior to any doctor, we believe that these ensemble results provide additional evidence to support the superiority of our AI model in diagnosing MFB and HC. We omitted the F1 score because it showed the same pattern as that of sensitivity and precision.

**Table 8:** Performance comparison between AI models with and without considering the ensemble technique in the external validation. The class-wise average and its standard deviation was given in measuring the sensitivity and precision of the ensemble AI.

Metrics	External validation results (%)
Accuracy of human residents	84.6 ±11.3
Accuracy of AI (without ensemble)	88.4 ±3.1
Accuracy of AI (with ensemble)	89.8
Sensitivity of human residents	82.9 ±9.7
Sensitivity of AI (without ensemble)	87.6 ±3.2
Sensitivity of AI (with ensemble)	88.3 ±6.9
Precision of human residents	82.9 ±8.8
Precision of AI (without ensemble)	85.2 ±4.6
Precision of AI (with ensemble)	88.1 ±6.5

### 3.3 Ablation study for the key-slice selector

In the previous section, we performed various experiments with the proposed approach to improve the classification and localization of MFBs or CRSs by using the selected key-slice set. In this section, we conduct additional experiments on the effect of key-slice selectors to provide further evidence of their originality.

By applying a simple key-slice detection of 2-D CNN (i.e., EfficientNet), we observed in the internal 5-folds cross-validation that the sub-slices including maxillary sinus were automatically extracted with an accuracy of 93.58 ±0.23%, demonstrating the excellent performance of our slice selector. Specifically, we compared the performance of the proposed key slice detector for various 2-D CNN models, as shown in Table 9. In order to prevent a potential overfitting issue due to learning with small data, a comparative experiment was performed with the version having the fewest parameters among the distributed versions of each model. In Table 9, the average performance results of all 2-D CNN models were similar within 1% accuracy and had a high accuracy of more than 93%. Therefore, we simply used EfficientNet, which is known as the latest among them, as a model for the key slice detector proposed.

**Table 9:** 2-D CNN performance comparison for detecting key-slices. The average and standard deviation were derived from each of 5-fold cross-validations.

Models	Internal validation accuracy
EfficientNet	93.58 ±0.23
VGG	93.45 ±0.15
DenseNet	93.36 ±0.09
ResNet	93.75 ±0.22
ResNext	93.42 ±0.30

To validate the effectiveness of the proposed key-slice selector, we also tested how the performance of the 3D-CNN (i.e., the second stage in the proposed AI system) changes with and without using the trained key-slice selector. When the key-slice selector was not used, all hemi-slices of each left- and right-sided 3D OMU CT were resized to 160 cubics (160 ×160 ×160) and used as input for the 3D-CNN. Table 10 shows this comparison result, where the 3-label (MFB, CRS, and HC) classification was evaluated in terms of micro and macro-average AUCs for the external validation. As this result shows, the key-slice selector increases the micro and macro-average AUCs by 1.4% and 2.0%, respectively. Although this performance improvement of 2% can be seen as a relatively small performance improvement, it reduces the error rate by 40%. This is because the key-slice selector reduced the error rate of the external validation from 5.1% to 3.1%, as shown in Table 10. This performance improvement prevented the AI system from diagnosing in areas other than the maxillary sinus, thereby validating the usefulness of our key-slice detector.

**Table 10:** 3-D CNN performance comparison of 3-label classification with and without using the proposed key-slice selector. The external validation was evaluated from each of the five pre-trained models.

Metrics	External validation results
Macro-average AUC (without key-slice selector)	0.949 ±0.005
Macro-average AUC (with key-slice selector)	0.969 ±0.005
Micro-average AUC (without key-slice selector)	0.952 ±0.009
Micro-average AUC (with key-slice selector)	0.966 ±0.005

### 3.4 External validation and performance evaluation through comparison with other algorithms and models

**Fig 4:**
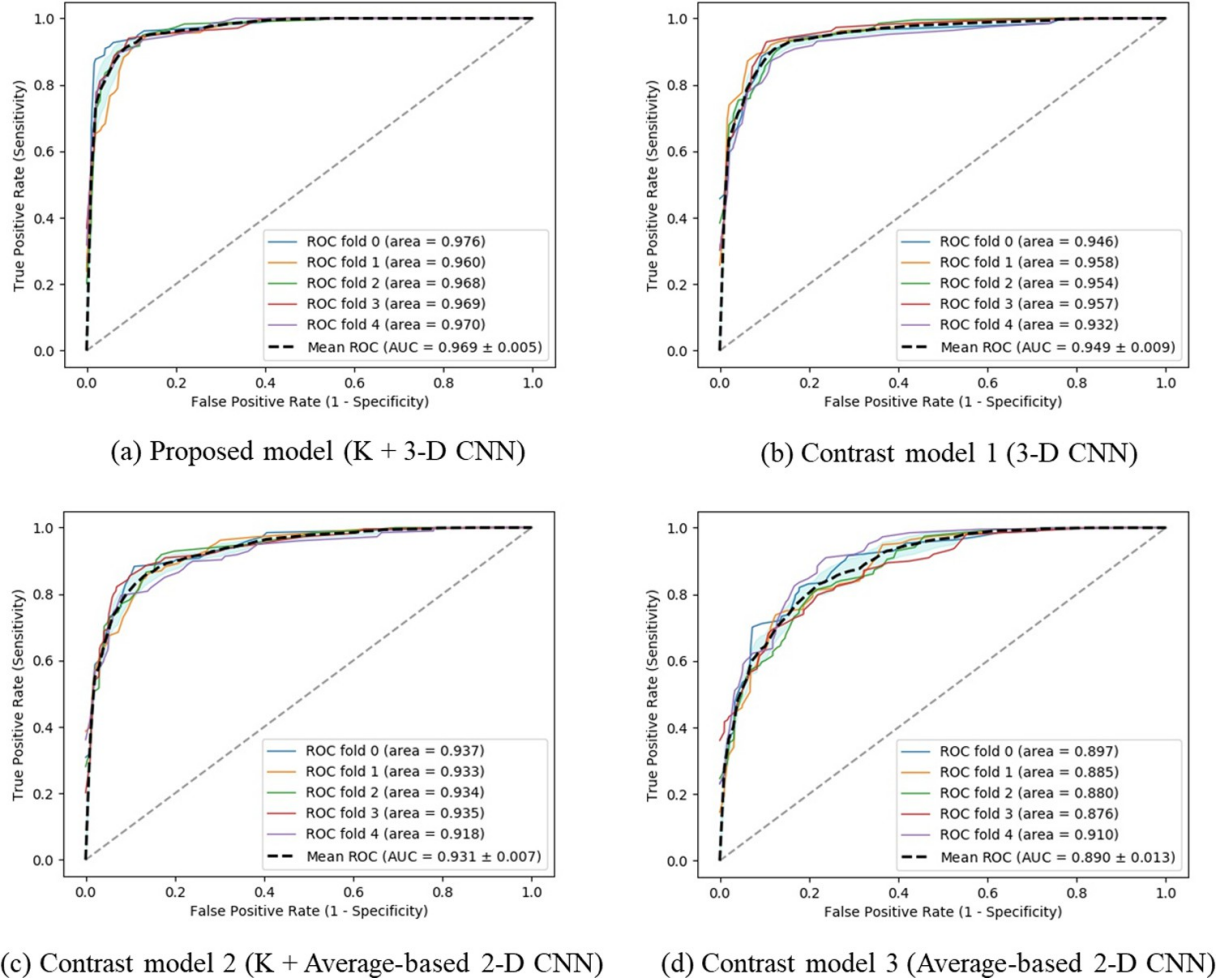
Performance comparison of the proposed scheme and the other configurations. The external validation results of macro-average AUCs were given from each of the five pre-trained models. K; the proposed key slice detector. Average-based 2-D CNN; 2-D CNN that takes the input as the average of 2-D coronal slices.

The proposed method sequentially combined 2-D CNN (key slice detector) and 3-D CNN (final disease classification) over two stages. To verify its originality, we performed comparative verification with other configurations, as shown in Fig 4. This figure showed the three contrast models: Contrast model 1 (Fig 4(B)) denotes the aforementioned model where the key slice detector is removed from the proposed method. Contrast model 2 (Fig 4(C)) denotes a model where the 3-D CNN used in the second stage in the proposed method is replaced by a 2-D CNN. By averaging coronal slices of 3-D input for the 3-D CNN used in the proposed method, we obtained a 2-D image used as input for the 2-D CNN in contrast to model 2. Like our key slice detector, the corresponding model also used EfficientNet. Contrast model 3 (Fig 4(D)) is where the proposed key slice detector is removed in contrast model 2. In each case with and without using a key slice selector at the first stage, we observed in Fig 4 that using 3-D CNN at the second stage increased macro-average AUC by more than 3% in comparison with using 2-D CNN at the second stage. In addition, in each case with and without using 3-D CNN at the second stage, we also observed that using a key slice selector improved macro-average AUC more than 2%. Considering both perspectives, we demonstrated that the combination (Fig 4(A)) of the proposed key slice detector and 3-D CNN-based disease classification shows the best performance compared to the algorithms (Fig 4(B)–4(D)) of other configurations. This verified the structural validity of the proposed technique compared to the other configurations/algorithms.

It is also worth comparing the performance of various base models for our 3-D CNN at the second stage. These external validation results are given in Table 11. As these results did not show a significant performance difference when using different 3-D CNNs, we performed all experiments by selecting ResNet18 as our 3-D CNN, with the smallest number of parameters.

**Table 11:** Performance comparison of 3-D CNN used for the second stage. The external validation was evaluated from each of the five pre-trained models.

Models	Macro- / Micro-average AUCs
ResNet18 (Ours)	0.969 ±0.005 / 0.966 ±0.005
ResNet34	0.967 ±0.008 / 0.962 ±0.008
DenseNet121	0.959 ±0.017 / 0.956 ±0.014

### 3.5 Qualitative analysis with visualizing the feature maps

**Fig 5:**
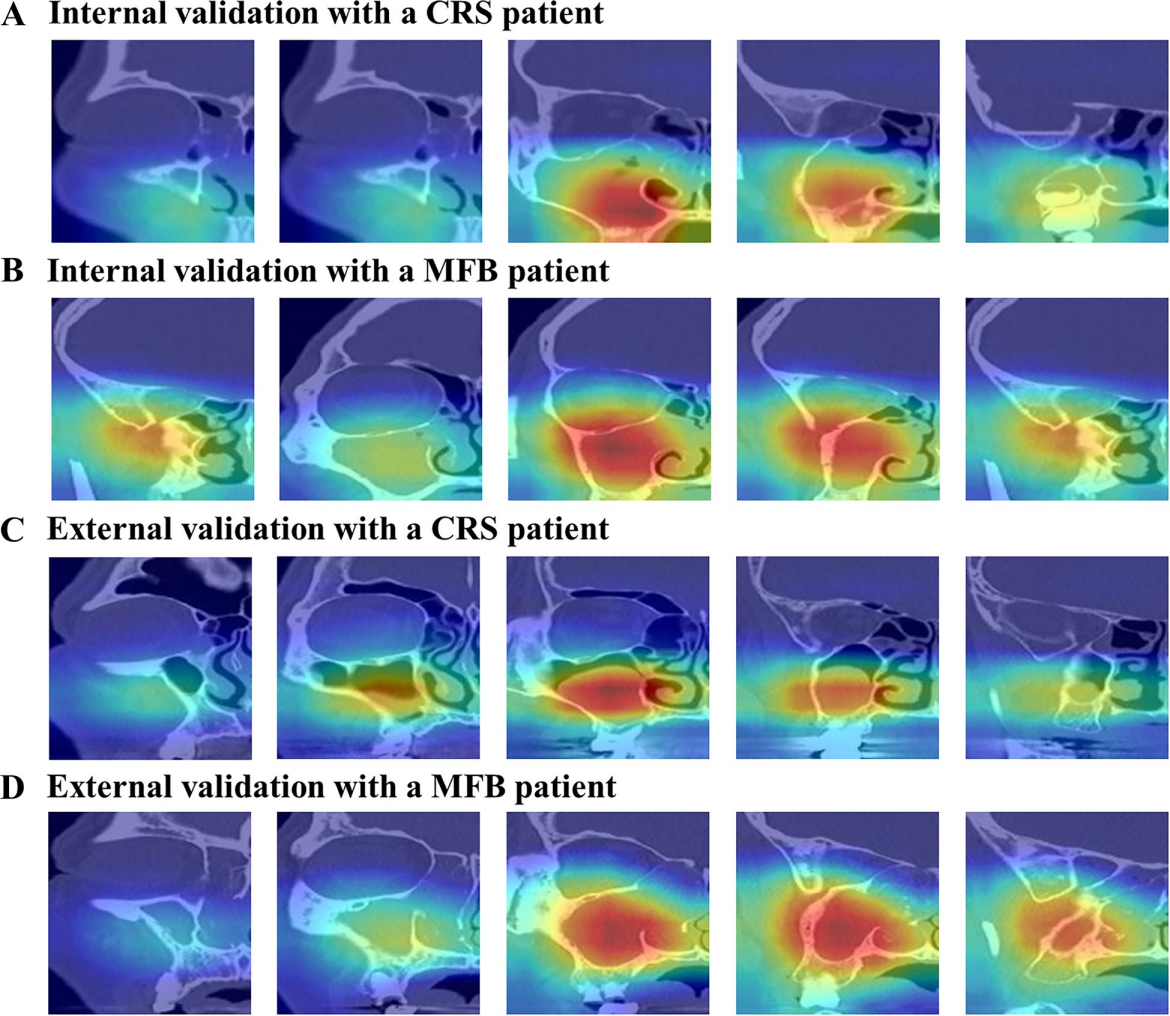
The internal (A and B) and external (C and D) validation results of the Grad-CAM of the patients with CRS and MFB.

Therefore, we have shown that AI has excellent diagnostic performance, but we did not sufficiently illustrate how these results were obtained. To present this, we used a technique called gradient-weighted class activation mapping (Grad-CAM) [13], which is useful in understanding which part of the input image led to the final classification decision of the target AI network. Fig 5 shows the visualization results for the internal and external datasets. This shows that there was a tendency to localize the partial areas of the maxillary sinus well.

In particular, compared to CRS, MFB is generally observed to have hyperdensity lesion inside the maxillary sinus or irregular surface margin. In other words, the shadow area inside the maxillary sinus can be regarded as the most critical imaging feature in MFB and CRS detections. As shown in Fig 5, the proposed AI classifier generally detected the entire inner surface of the maxillary sinus of a sample and was mainly activated on the area of soft tissue density in this surface. In other words, these results indicate that AI also views the most important feature of disease classification like radiologists, supporting the validity/interpretability of the high diagnostic results of AI. It is also helpful to note that our study only exploited the information that the maxillary sinus was visible in a specific coronal 2-D slice but did not utilize any information for the maxillary sinus’s location on each coronal 2-D slice in training the network. From this point of view, AI has learned the disease location in the maxillary sinus by itself, demonstrating that these AI results were not accidental.

## 4 Discussion

A CT scan is the most common and essential tool for diagnosis among the tests available as a tool for preoperative evaluation of endoscopic sinus surgery. The most representative finding of a fungal ball in a CT scan is intralesional calcification or metallic densities [14, 15]. Calcification or metallic densities in the maxillary sinus are thought to arise from metal ions deposited in the necrotic area of the mycelium [16]. However, when zinc deficiency is present, intracellular storage is reduced due to homeostasis [17], sinonasal calcifications or metallic densities may not be visible. In previous studies, hyperdense lesions inside the fungal ball were observed in 80–82.8% [18, 19] and had a high specificity (99–100%) [7, 20]. Other common CT findings include unilateral involvement, bony thickening of the involved sinus wall, total haziness of the sinus with mass effect, and irregular surface of the materials [7, 21]. Bony erosion or thickening of the involved sinus is associated with the degree of chronic inflammation of the mucosa surrounding the fungal ball [22]. When performing endoscopic sinus surgery, a dirty, clay-like appearance can be viewed as typical findings of mycetoma, and these findings can reveal the spiculated surface on the CT scan.

Therefore, the detection and differentiation of fungal balls through a CT scan are essential for determining further treatment strategies. However, general physicians, not otolaryngologists or radiologists, cannot distinguish maxillary sinus fungal balls on CT scans. To solve this problem, deep learning using the CNN algorithm shows excellent potential for medical image-based analysis. Deep learning is a type of machine learning that includes convolutional layers, fully connected layers, and an output layer with the help of neural networks, images, videos, and unstructured data that can be analyzed with the less human intervention [23]. The difference from machine learning is that deep learning algorithms use the original data to define the representations for classification, similar to the human brain. In many previous studies, CNNs have performed the image-based analysis with accuracy similar to that of physicians. Studies have performed deep learning techniques using plain radiographs to detect and classify maxillary sinusitis [24–26]. When only the Waters’ view radiograph was used, the diagnostic performance was approximately 0.88–0.94 AUC [25], and when a multi-view model was made with Waters’ and Caldwell view radiographs, it showed a higher AUC than the single Waters’ view model [26]. In addition, in a study of MRI-based machine learning techniques, Fujima *et al*. investigated the predictive power of the treatment outcome of sinonasal squamous cell carcinomas. The validation dataset was able to predict local control and failure with an accuracy of 0.92 [27]. In another study, Ramkumar *et al*. reported that AI could differentiate squamous cell carcinomas from sinonasal IP through MRI-based texture analysis, showed an accuracy of 89.1%, and did not significantly differ from the neuroradiologist’s review (87.0%) [28].

There have been three studies on CT-based image analysis using CNN in the rhinology area. Huang *et al*. performed supervised analysis through a coronal sinus CT scan to classify the location of the anterior ethmoidal artery and reported an accuracy of 82.7% and AUC of 0.86 using 675 images [3]. One slice was selected to represent the anterior ethmoidal foramen, and the lamina papyracea, anterior skull base, and middle turbinate were included in the image, and the contralateral hemi-slice was flipped on the vertical axis for consistent training data. In the aforementioned study, two ENT residents trained the AI after classifying the anterior ethmoidal location using CT scans. However, training using histopathologically confirmed fungal balls is likely to result in more minor training errors in our study. Parmar *et al*. were able to classify the existence of concha bullosa, which could be missed from the checklist when performing sinus surgery using a CNN (Inception-V3) [4]. A total of 447 coronal CT slice images were used, with a diagnostic accuracy of 81% and an AUC of 0.93.

Similarly, in a previous study, some false-negative cases may occur when using only one coronal CT slice. Chowdhury *et al*. revealed the classification of osteometal complex occlusion in CRS patients using a 2-D CNN (Inception-V3) [5]. Their results showed 85% classification accuracy and an AUC of 0.87. The aforementioned three studies used one 2-D CT slice, which does not reflect how trained real-world physicians analyze.

Recently, 3-D CNNs have been developed to preserve the 3-D context of CT images composed of successive sequence slices since 3-D CNNs can better capture spatial information and extract more real features [29]. In particular, a computer-aided diagnosis (CAD) system using 3-D CNN to detect and classify lung cancer or pulmonary nodules has been developed using CT images in several studies [6, 30, 31]. These studies revealed that 3-D CNN has advantages over 2-D CNN and that deep 3D CNN can improve the performance of CAD systems. While this study has been limited to a binary classification between CRS and HC groups, we have solved the more difficult problem of simultaneously discriminating CRS and MFB against HC (i.e., 3-label classification). In our study, as the proposed AI algorithm used a whole stack of CT images via the 3-D CNN, image classification was possible by additionally exploiting information on adjacent associations between slices. Therefore, as it can effectively detect subtle differences (i.e., correlation) between adjacent coronal slices, our algorithm was able to produce a superior result compared to previous studies with AUC 0.97 in this 3-label classification. In addition, as we introduced in Section 3.3, we applied a novel key-slice detection method to further optimize the generalization performance for a given limited number of training samples. As the sub-slices, including the maxillary sinus, were automatically extracted, the classification error was reduced by 40%, supporting the originality and validity of our AI system.

The 2-D CNN and 3-D CNN used in our algorithm were pre-trained using ImageNet and Kinetics datasets, respectively, as these datasets have various classes and a sufficient number of samples for each class. Therefore, these datasets are suitable as data for pre-training AI. However, they are not medical images, probably reducing the performance of fine-tuning with our medical data. If a large set of 2-D or 3-D medical images (e.g., OMU CTs) with various classes can be used for pre-training our AI system in the future, we expect its diagnostic performance to be further improved. It is also helpful to note that our AI system did not use any annotation (e.g., segmentation or localization) for disease location. Even though this annotation was not performed, our system successfully predicted the location of the target disease, as shown in Section 3.4. Nevertheless, localization can be more accurate with less training data if the corresponding annotation is utilized. We will proceed with these studies in future work.

**Fig 6:**
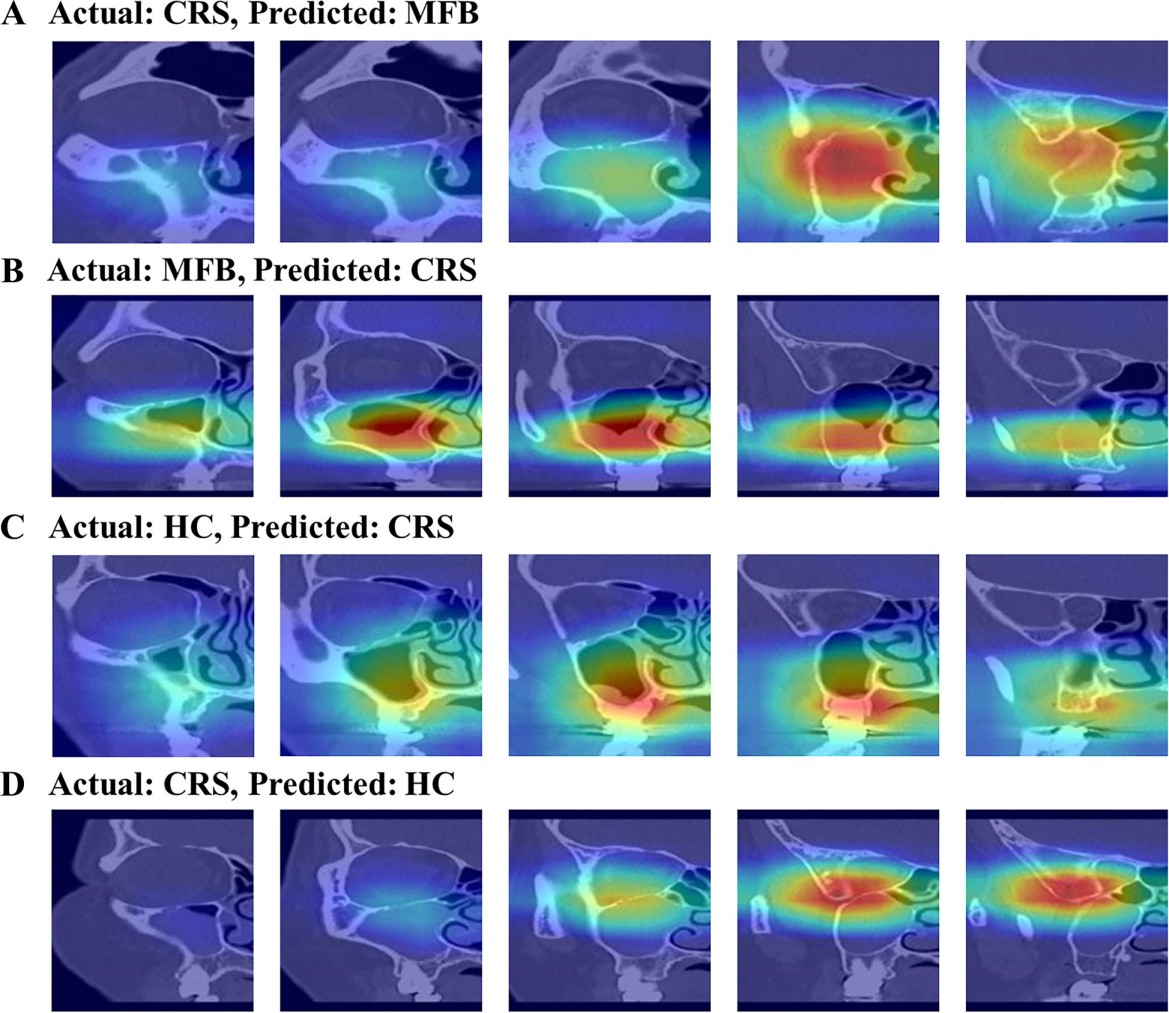
Examples of the Grad-CAM misclassified by the AI system on the external validation. MFB; maxillary sinus fungal ball, CRS; chronic rhinosinusitis, HC; healthy control.

We illustrated in Fig 6 some examples of misclassified images for external validation to obtain some insight into the network’s logic. We excluded cases of misclassification between MFB and HC. It rarely happened (e.g., it happened once at most in our external validation, making it difficult to discuss the cause of the error). In both examples of AI predicting CRS as MFB or MFB as CRS in Fig 6A and 6B, the AI correctly chose a location with abnormality. However, it is difficult to differentiate these two cases radiologically because of the full haziness of the unilateral maxillary sinus (Fig 6A) or polypoid mucosal surface (Fig 6B). In the example of AI predicting HC as CRS in Fig 6C, the image was misdiagnosed as abnormal by focusing on the area with minimal mucosal swelling at the inferolateral part of the maxillary sinus. In other words, this example is a case clinically suitable for HC and labeled as HC by an experienced otolaryngologist. However, it can be classified as CRS in the radiological aspect. Therefore, this example provides a rationale for certain AI misclassifications. In the example of AI predicting CRS as HC in Fig 6D, the AI-focused on the outside of the maxillary sinus, which led to incorrect classification. We could not determine why the AI-focused elsewhere, which is considered the limit of deep learning methods. Nevertheless, we believe that these low-probability misclassifications can be further improved by adopting more learning data and by further advancing AI technology.

## 5 Conclusion

The proposed system is the first fully automated MFB (or CRS) recognition algorithm that utilizes a deep learning approach in OMU CT to the best of our knowledge. Our novel method automatically selected key sub-slices with the presence of maxillary sinus in the first stage using 2-D CNN, robustly distinguished MFB, CRS, and HC, in the second stage using 3-D CNN and finally localized the target disease area. Our model achieved a high AUC value of 0.97 and a higher accuracy of 88.4% than the 84.6% of the otolaryngology majors with lower variance. We believe our AI approach will facilitate diagnostic inspection and provide a useful diagnostic inspection screening tool for the region where otolaryngology specialists are scarce.
